# Childhood unpredictability, expressive suppression, and mindfulness as predictors of bystanders' outsider behaviors in cyberbullying among Chinese undergraduates: a moderated mediation model

**DOI:** 10.3389/fpsyg.2026.1798978

**Published:** 2026-03-25

**Authors:** Rui Chen, Dan Ming, Ting Zhou, Xin Hao, Cui-ying Fan

**Affiliations:** 1 School of Medical Humanities, Hubei University of Chinese Medicine, Wuhan, China; 2 Hubei Shizhen Laboratory, Wuhan, China; 3 Hubei Health Industry Development Research Center, Wuhan, China; 4 Research Institute of Nuclear Power Operation, Wuhan, China; 5 School of Education and Sports Science, Yangtze University, Jingzhou, China; 6 Key Laboratory of Adolescent Cyberpsychology and Behavior (CCNU), Ministry of Education, School of Psychology, Central China Normal University, Wuhan, China

**Keywords:** bystander, childhood unpredictability, cyberbullying, expressive suppression, mindfulness, undergraduates

## Abstract

**Background:**

Bystanders' outsider behaviors play a vital role in the development and persistence of cyberbullying incidents, a prevalent form of harmful digital engagement. Prior literature has found an association between childhood unpredictability and bystanders' outsider behaviors in cyberbullying. However, the underlying mechanisms explaining this association remain unclear. To address this, the current study examined the mediating role of expressive suppression and the moderating role of mindfulness between childhood unpredictability and bystanders' outsider behaviors in cyberbullying.

**Methods:**

A cross-sectional survey was conducted with a total of 1,149 Chinese undergraduates (*M*_*age*_= 19.48 years, *SD*_*age*_= 2.79, age range: 18–24 years) recruited through a convenience sampling method. Well-validated self-report measures were administered to assess childhood unpredictability, expressive suppression, mindfulness, and bystanders' outsider behaviors in cyberbullying. Structural equation modeling (SEM) was used to test a moderated mediation model in which expressive suppression mediates the association between childhood unpredictability and bystanders' outsider behaviors in cyberbullying, with mindfulness moderating the relationship between expressive suppression and bystanders' outsider behaviors in cyberbullying.

**Results:**

The study revealed that childhood unpredictability was significantly and positively associated with bystanders' outsider behaviors in cyberbullying among undergraduates. Mediation analysis demonstrated that childhood unpredictability both directly predicted bystanders' outsider behaviors in cyberbullying and indirectly affected them through the mediating role of expressive suppression. Furthermore, the mediating effect of expressive suppression was moderated by mindfulness. Specifically, undergraduates with lower mindfulness showed a stronger association between expressive suppression and bystanders' outsider behaviors in cyberbullying compared to those with higher mindfulness.

**Conclusion:**

This study enriches existing literature by elucidating that the mediating effect of expressive suppression and the moderating effect of mindfulness represent additional explanations of the association between childhood unpredictability and bystanders' outsider behaviors in cyberbullying among undergraduates. The findings offer empirical evidence for developing targeted interventions aimed at reducing outsider behaviors in cyberbullying and promoting a healthier online environment, thereby contributing to the psychological wellbeing of undergraduates within emerging digital environments.

## 1 Introduction

The proliferation of the Internet and information technologies has increased the prevalence of cyberbullying among undergraduates (Wang et al., 2023; Li and Xia, 2024; Tong et al., 2024; Tian et al., 2025). Cyberbullying is defined as intentional and repeated aggression by individuals or groups via electronic communication tools, often targeting victims who are unable to defend themselves effectively (Smith et al., 2008). Several distinctive features of digital environments characterize it, including temporal asynchrony, spatial transcendence, self-disinhibition, and interpersonal anonymity (Zhang et al., 2023). Undergraduates are particularly susceptible to cyberbullying involvement, a vulnerability often attributed to their extensive digital engagement, limited external oversight, and heightened propensity for online self-disclosure (Luo et al., 2023; Li and Xia, 2024). Previous studies indicate that approximately 59% to 70% of undergraduates have witnessed cyberbullying on social media platforms (Gahagan et al., 2016; Doane et al., 2020). In China, the prevalence of cyberbullying is also as high as 64% among undergraduates (Huang et al., 2021). Research has shown that individuals involved in cyberbullying, whether as victims or perpetrators, are at heightened risk for a range of adverse psychological and behavioral outcomes, such as depression, anxiety, and suicidal ideation (Doane et al., 2014; Bussey et al., 2015; Kwan et al., 2020; Guo and Xia, 2023; Kasturiratna et al., 2025). Thus, the high prevalence and severe consequences of cyberbullying underscore the need to understand the complex dynamics of these online interactions within the broader landscape of digital engagement and to develop effective interventions aimed at safeguarding psychological wellbeing.

To effectively address this substantial challenge, it is crucial to deconstruct the social dynamics underlying cyberbullying, which is widely conceptualized not as a dyadic interaction but as a group process involving three distinct roles: perpetrators, victims, and bystanders (Kowalski et al., 2014). Bystanders refer to individuals who witness or become aware of cyberbullying incidents without being direct perpetrators or targets (Stanciu and Chis, 2021). Owing to the inherent anonymity and accessibility of cyberspace, bystanders, as the majority group in such incidents, play a critical yet often neglected role in the dynamics of cyberbullying (Chen, 2024; Knauf and Eschenbeck, 2025). A longitudinal study among Malaysian youth supports their numerical dominance, reporting that the proportion of bystanders rose from 61% to 70% over 2 years (Balakrishnan, 2015, 2017). Furthermore, scholars argue that bystanders' decisions about whether and how to intervene can decisively influence the escalation, persistence, and resolution of cyberbullying incidents, with significant consequences for both victims and perpetrators (Macaulay et al., 2022). For instance, Bystanders may offer direct or indirect support to alleviate victims' distress or, conversely, reinforce perpetrators' actions and amplify harm (Bastiaensens et al., 2014; Chen et al., 2025). Notably, in cyber contexts, such consequences are heightened by the public, permanent, and searchable nature of digital content; a single supportive comment can provide lasting comfort, while passive inaction can amplify harm by signaling social acceptance to a wide audience (Hinduja and Patchin, 2022). However, empirical evidence suggests that most bystanders remain passive, typically engaging in non-intervention through passive observation or active disengagement from cyberspace (Bastiaensens et al., 2014; Gahagan et al., 2016). This active disengagement includes uniquely digital behaviors such as scrolling past incidents, muting notifications, or exiting the platform entirely, actions that collectively contribute to a climate of indifference (Shen et al., 2024). Song and Oh (2018) clearly quantified this trend, finding that 69.4% of bystanders took no action upon witnessing cyberbullying. Such individuals correspond to the established category of the outsider role within dominant bystander typologies, defined by a non-interventionist stance manifested as either passive observation or active avoidance of incidents (Shen et al., 2024; Chen et al., 2025). More critically, far from being neutral, such inaction implicitly endorses perpetrators' aggression, legitimizing their conduct and exacerbating psychological harm to victims (Hinduja and Patchin, 2022; Shen et al., 2024). Despite the profound negative impact of this dominant bystander response, existing research disproportionately emphasizes the antecedents and outcomes of intervention behaviors, while systematic investigation into outsider behavior in cyberbullying dynamics remains limited and largely qualitative (Dang and Liu, 2022). Given bystanders' substantial presence and critical function in cyberbullying, researchers must move beyond descriptive accounts and systematically investigate the psychological factors that predispose individuals to adopt this passive, yet harmful, bystander stance.

A further limitation of prior literature is its narrow temporal focus. Specifically, studies examining antecedents of bystander behavior have largely centered on contemporaneous experimental, psychological, and situational factors, leaving a systematic explanatory framework that integrates a developmental perspective notably underdeveloped (Qi et al., 2024). Informed by this perspective, childhood unpredictability, which refers to an individual's perception of disorder and variability in the early life environment and caregiver behaviors, has been identified as a critical yet frequently overlooked distal factor (Doom et al., 2017; Wang et al., 2022; Maner et al., 2023). According to life history theory (Hill, 1993), exposure to highly unpredictable early environments shapes individuals' behavioral strategies, promoting a fast life history strategy characterized by immediate gratification, risk aversion, and discounting of future rewards (Griskevicius et al., 2011; Wang et al., 2022). A growing body of research suggests that such early environmental risks may shape behavioral tendencies in ambiguous social contexts (Doom et al., 2017; Feng et al., 2024), such as cyberbullying incidents. Although prior literature has indicated that early life ecological risks may affect individuals' cyberbullying behaviors, the specific mechanisms through which early life unpredictability translates into outsider bystander role remain poorly understood (Li et al., 2024, 2025). Clarifying this association is therefore crucial for developing effective bystander-focused interventions in cyberbullying. Accordingly, this study focuses on bystanders' outsider behaviors and examines the association between childhood unpredictability and such behaviors, with the ultimate aim of informing the design of constructive, bystander-oriented interventions. By guiding bystanders toward more positive roles, this research seeks to mitigate the prevalence and detrimental effects of cyberbullying among undergraduates.

Childhood unpredictability may play a vital role in bystanders' outsider behaviors among undergraduates. According to life history theory (Hill, 1993), a valuable theoretical framework for explaining this relationship, early experiences could shape individuals' adaptive behavioral strategies. Specifically, the theory elucidates that the unique conceptual basis of childhood unpredictability lies in positing that its core element, perceived environmental randomness and uncertainty, calibrates life history strategies in a distinct manner, and in turn, predisposes individuals to specific bystander responses (Ellis et al., 2009; Luo et al., 2020; Xu et al., 2023). Individuals exposed to high levels of unpredictability in childhood, such as frequent changes in family structure, inconsistent caregiving, or chaotic daily routines, tend to develop a fast life history strategy. This strategy prioritizes immediate safety and minimizes investment in long-term or uncertain social interactions (Lindert et al., 2022; Wang et al., 2022). This strategic adaptation directly affects social cognition, leading individuals to perceive ambiguous social contexts, such as cyberbullying incidents, as unpredictable and uncontrollable (Szepsenwol et al., 2022; Qi et al., 2024). Consequently, their propensity for active engagement or intervention is fundamentally diminished.

Moreover, individuals who experienced high childhood unpredictability often develop a sense of helplessness or detachment toward social conflicts as a result of chronic instability (Szepsenwol et al., 2022). They may acquire a learned avoidance of investing in social interactions that lack predictability. Such investments are perceived to carry uncertain risks, such as retaliation from bullies or failed intervention attempts, while offering no guaranteed benefits, including successful victim protection or tangible situation improvement (Wang et al., 2022; Topel et al., 2023; Park et al., 2024). This tendency to disengage from unpredictable social contexts directly extends to cyberbullying scenarios. When witnessing cyberbullying, those individuals may perceive intervention as either risky or futile, which leads them to adopt characteristic outsider behaviors, such as ignoring the incident or consciously avoiding involvement, to preserve their emotional and social safety (Doom et al., 2024; Qi et al., 2024). Similarly, Szepsenwol et al. (2022) found that high childhood unpredictability was associated with lower emotional control and reduced investment in social relationships, both linked to passive responses in interpersonal conflicts. Wang et al. (2022) also reported that childhood unpredictability predicted a preference for intuitive, low-effort decision-making under uncertainty. This preference manifests in strategies such as disengagement from complex social situations, including cyberbullying.

Additionally, childhood unpredictability may disrupt the development of prosocial tendencies and a sense of social responsibility (Lindert et al., 2022). A fast life history strategy disfavors long-term social investments in environments where future returns are uncertain (Lindert et al., 2022; Topel et al., 2023). Individuals raised in unpredictable environments may have fewer opportunities to learn consistent social norms, such as helping behavior, or to build trust in the effectiveness of social intervention. Their early experiences teach them that social support and stable relational outcomes are unreliable (Doom et al., 2024; Qi et al., 2024). When acting as bystanders in cyberbullying, this lack of prosocial conditioning and trust in social systems further reinforces outsider behaviors. These individuals may fail to recognize the impact of their inaction on the victim, or they may doubt that their intervention will lead to any positive change (Szepsenwol et al., 2022; Topel et al., 2023; Li et al., 2025). Empirically, studies have linked childhood unpredictability to reduced prosocial behavior in peer contexts (Lindert et al., 2022; Wang et al., 2022; Xu et al., 2023), a pattern that is likely to extend to cyberbullying bystander roles. For instance, Lindert et al. (2022) found that childhood unpredictability predicted lower engagement in supportive social behaviors, even in low-risk online settings. Although direct evidence linking childhood unpredictability to cyberbullying bystander behavior is limited, indirect support derives from its established association with reduced prosociality and social engagement, which are key antecedents of bystander intervention (Lindert et al., 2022; Wang et al., 2022; Doom et al., 2024). Based on the theoretical and empirical evidence, it is reasonable to hypothesize that:

**Hypothesis 1 (H1):** *Childhood unpredictability is positively associated with bystanders' outsider behaviors in cyberbullying among undergraduates*.

Despite accumulating evidence linking childhood unpredictability to bystanders' outsider behaviors in cyberbullying, the psychological mechanism underlying this association remains unclear (Koss et al., 2025). Previous studies have suggested that early unpredictable environments, such as household chaos and unstable caregiving, can disrupt the development of adaptive emotion regulation strategies. These strategies, in turn, shape individuals' social response patterns, including behaviors in cyberbullying (Wade et al., 2022; Chu et al., 2023; Meyer et al., 2025). Integrating life history theory and the modal model of emotion regulation, we posit that expressive suppression may serve as a critical mediator linking childhood unpredictability to bystanders' outsider behaviors in cyberbullying (Turliuc et al., 2020; Meyer et al., 2025). As a response-focused emotion regulation strategy, expressive suppression involves actively inhibiting emotional expressions. This strategy often fails to alleviate negative affect and instead impairs social functioning by limiting meaningful emotional engagement with others (Turliuc et al., 2020; Meyer et al., 2025). Individuals who rely on expressive suppression tend to avoid outwardly acknowledging emotions, thereby reducing their willingness to intervene in social conflicts or support others. These outcomes reflect key features of outsider behaviors in cyberbullying (Weissman et al., 2019; Chu et al., 2023; Kuhlman et al., 2023). Given that childhood unpredictability undermines the learning of flexible emotion regulation, individuals exposed to such environments may over-rely on suppression to cope with uncertainty, thereby further reinforcing passive social responses (Wade et al., 2022; Warmingham et al., 2023).

Childhood unpredictability may be closely associated with the tendency to use expressive suppression (Schäfer et al., 2017; Gross and Cassidy, 2019; Szepsenwol et al., 2022). A longitudinal study supports this view, demonstrating that household chaos, a core indicator of childhood unpredictability, predicts greater use of expressive suppression during adolescence (Meyer et al., 2025). This link arises because unstable early environments limit opportunities to learn adaptive emotion regulation while simultaneously promoting emotion inhibition as a learned safety seeking strategy to navigate unpredictability (Wade et al., 2022; Warmingham et al., 2023). For instance, individuals from chaotic households often report suppressing emotional reactions to avoid conflict or unpredictability in caregiving, a pattern that persists into social interactions such as cyberbullying (Hong et al., 2018; Chu et al., 2023). At a systemic level, chronic unpredictability calibrates stress-response systems to prioritize short-term stability over long-term social investment (Wade et al., 2022; Radley and Herman, 2023). Consequently, when faced with ambiguous social cues such as those in cyberbullying, expressive suppression becomes a default strategy for managing overwhelming emotions, thereby reinforcing a passive bystander stance (Weissman et al., 2019; Meyer et al., 2025). Beyond shaping long-term behavioral strategies, childhood unpredictability also heightens vulnerability to immediate social stress by eroding emotional resilience (Chu et al., 2023; Buthmann et al., 2024). This erosion reduces the ability to adaptively cope with subsequent social challenges, including the stress of witnessing cyberbullying, and instead increases reliance on expressive suppression as a compensatory mechanism (Weissman et al., 2019; Warmingham et al., 2023). As these suppression tendencies deepen and consolidate through repeated use in response to ongoing social stressors, they evolve into a stable, automatic response pattern that fundamentally shapes how individuals engage with or disengage from social conflicts, further solidifying the positive association between childhood unpredictability and expressive suppression (Weissman et al., 2019; Turliuc et al., 2020).

Beyond serving as an outcome of early experience, expressive suppression is theorized to directly facilitate bystanders' outsider behaviors in cyberbullying (Weissman et al., 2019; Chu et al., 2023; Da Silva Gomes et al., 2024). The ambiguous social cues characteristic of cyberbullying, such as anonymous perpetrators and uncertain harm, require emotional awareness and proactive involvement for effective resolution (Turliuc et al., 2020; Chu et al., 2023). In contrast, expressive suppression impedes the processing of and response to others' emotions. This regulatory mechanism reduces attentional allocation to a victim's distress signals and undermines motivation to intervene, as recognizing such emotions would compel individuals to confront their own suppressed affective states (Turliuc et al., 2020; Meyer et al., 2025). This mechanistic account of disengagement is deepened by considering the temporal perspective inherent in emotion regulation. Socioemotional selectivity theory posits that suppression reflects a focus on immediate emotional management rather than long-term social investment (Meyer et al., 2025). This narrowed time horizon makes individuals more likely to engage in outsider behaviors, as proactively supporting a victim represents an investment they are reluctant to make (Charles and Carstensen, 2008; Chu and Carstensen, 2023; Meyer et al., 2025). Empirical evidence supports this pathway, indicating that expressive suppression predicts bystander responses in cyberbullying. Individuals who rely more heavily on suppression are significantly more likely to remain passive observers and less likely to intervene or support victims (Weissman et al., 2019; Chu et al., 2023). Moreover, reliance on suppression is associated with emotional disconnection from such incidents, which impedes recognition of the consequences of inaction and thereby reinforces outsider behaviors (Da Silva Gomes et al., 2024; Ye et al., 2024). Thus, childhood unpredictability reinforces expressive suppression, which in turn reduces the likelihood of active bystander behavior. Therefore, we hypothesize that:

**Hypothesis 2 (H2):** *Expressive suppression mediates the association between childhood unpredictability and bystanders' outsider behaviors in cyberbullying among undergraduates, such that (a) higher levels of childhood unpredictability predict higher levels of expressive suppression, and (b) higher expressive suppression predicts higher levels of bystanders' outsider behaviors in cyberbullying*.

While the aforementioned mediation pathway outlines a potential mechanism linking childhood unpredictability to bystander outsider behaviors via expressive suppression, individual responses inevitably vary. Notably, not all individuals with a heightened tendency for expressive suppression subsequently adopt a passive bystander stance. This variability points to the potential involvement of protective factors that may buffer the effects of expressive suppression on bystanders' outsider behaviors in cyberbullying among undergraduates. Identifying such protective factors is crucial for developing nuanced interventions.

Research increasingly highlights the pivotal role of mindfulness in regulating emotional and behavioral responses to interpersonal stressors, including those encountered in cyberspace (Chen et al., 2024; Bussey and Luo, 2024; Khoury and Vergara, 2025; Golchoobi and Nooripour, 2025). Mindfulness, defined as purposeful and non-judgmental attention to present-moment experiences, fosters the ability to observe internal states such as suppressed emotions without immediate reactivity or avoidance (Wunder and Jones, 2023; Pruessner et al., 2024; Sparacio et al., 2024; Lo, 2024). Grounded in established theoretical frameworks, mindfulness facilitates disengagement from rigid, automatic response patterns when confronting emotional conflicts (Rowland and Wenzel, 2020; Shankland et al., 2021). Within the specific demands of a cyberbullying incident, this entails managing the tension between the habitual suppression of negative emotions and the social-moral pressure to intervene (Carpenter et al., 2019; Prieto-Fidalgo et al., 2022). Rather than being overwhelmed by suppressed emotions that typically drive passive behaviors, individuals with a mindful approach can adopt a more flexible perspective. They learn to decenter from these emotions, perceiving them as transient mental events rather than fixed imperatives for action or withdrawal (Bussey and Luo, 2024).

Beyond its foundational role in fostering present-moment awareness, empirical evidence directly supports mindfulness as a buffer in the pathway from suppression to behavior. It enhances emotional awareness and reduces reliance on unconscious suppression (Carpenter et al., 2019; Wunder and Jones, 2023; Sparacio et al., 2024). For individuals prone to high expressive suppression, mindfulness attenuates the link between habitual suppression and its typical maladaptive behavioral outcomes (Guendelman et al., 2017). A key mechanism underlying this buffering effect is the cultivation of emotion regulation flexibility. For instance, mindfulness training significantly reduces rumination, a common cognitive consequence of suppression, while enhancing the flexibility of emotional responses, even among vulnerable populations (Wunder and Jones, 2023; Sparacio et al., 2024). Within cyberbullying contexts, this cultivated flexibility may reduce the likelihood of undergraduates adopting bystanders' outsider behaviors, such as ignoring incidents or remaining silent, when confronted with suppressed negative emotions including anxiety or guilt (Aldao et al., 2010; Sheppes et al., 2015; Lv et al., 2021; Prieto-Fidalgo et al., 2022). Instead of impulsive avoidance, the enhanced executive control associated with mindfulness enables more constructive appraisal of the situation and a greater capacity to consider proactive responses (Gallant, 2016; Bussey and Luo, 2024).

In addition to promoting cognitive and behavioral flexibility, mindfulness may buffer the effects of suppression through non-judgmental acceptance of internal experiences. This acceptance allows individuals to experience thoughts and emotions without attempting to alter or judge them (Lindsay and Creswell, 2019; Wunder and Jones, 2023; Wenzel et al., 2024). When undergraduates suppress emotions related to cyberbullying, such as fear of retaliation or uncertainty about intervention, they often experience significant inner conflict (Peters et al., 2014; Sahib et al., 2023; Chu et al., 2023). If unmanaged, this conflict escalates into experiential avoidance, which directly manifests as the withdrawal or indifference characteristic of outsider behaviors (Hayes et al., 2006; He et al., 2024). Mindfulness may help reduce this conflict by encouraging acceptance of difficult emotions without self-criticism (Golchoobi and Nooripour, 2025). For instance, a mindful individual may acknowledge feelings of uncertainty about how to help without translating that uncertainty into a negative self-judgment of incompetence, thereby reducing the likelihood of complete situational avoidance (Junça-Silva and Caetano, 2023). Furthermore, this non-judgmental stance cultivates self-compassion, which serves as an additional protective factor against the passive responses typically driven by emotional suppression (Marshall et al., 2020; Wunder and Jones, 2023; Sparacio et al., 2024).

Mindfulness may also exert its protective influence by restoring a sense of personal control. Undergraduates prone to high expressive suppression often experience a paralyzing sense of powerlessness over their own emotions and cyberbullying incidents, which directly reinforces outsider behaviors (Garland, 2024). Mindfulness counteracts this by enhancing perceived control over one's emotional and behavioral responses (Hoge et al., 2021; Golchoobi and Nooripour, 2025). A present-moment focus displaces rumination on past failures or future fears. By anchoring awareness in the present, highly mindful individuals can more clearly assess their capacity to act, even amidst ambiguity. This present-centered appraisal fosters greater intervention self-efficacy, or the belief that one's actions can make a difference (Sharma and Kumra, 2022). This aligns with findings by Bussey and Luo (2024), who demonstrated that mindfulness moderated the link between moral disengagement and cyberbullying perpetration. Youth with higher mindfulness were less likely to justify inaction. Thus, this mindfulness-enhanced sense of control translates into distinct bystander behavior patterns. Empirical evidence consistently associates higher mindfulness with reduced passive bystanding and increased active intervention (Malin and Gumpel, 2023).

Given these theoretical and empirical evidences, mindfulness could be hypothesized to moderate the relationship between expressive suppression and undergraduates' outsider behaviors in cyberbullying. Specifically, higher mindfulness levels will alleviate the positive association between expressive suppression and outsider behaviors, such that undergraduates with high expressive suppression and high mindfulness could engage in fewer outsider behaviors than those with high expressive suppression and low mindfulness. Conversely, lower mindfulness might amplify the effect of expressive suppression, as undergraduates lack the emotional flexibility and control to counteract the passive tendencies driven by suppressed emotions. Accordingly, we hypothesize that:

**Hypothesis 3 (H3):** *Mindfulness moderates the strength of the positive association between expressive suppression and undergraduates' outsider behaviors in cyberbullying, such that this positive association is weaker among undergraduates with higher levels of mindfulness*.

**Figure 1:**
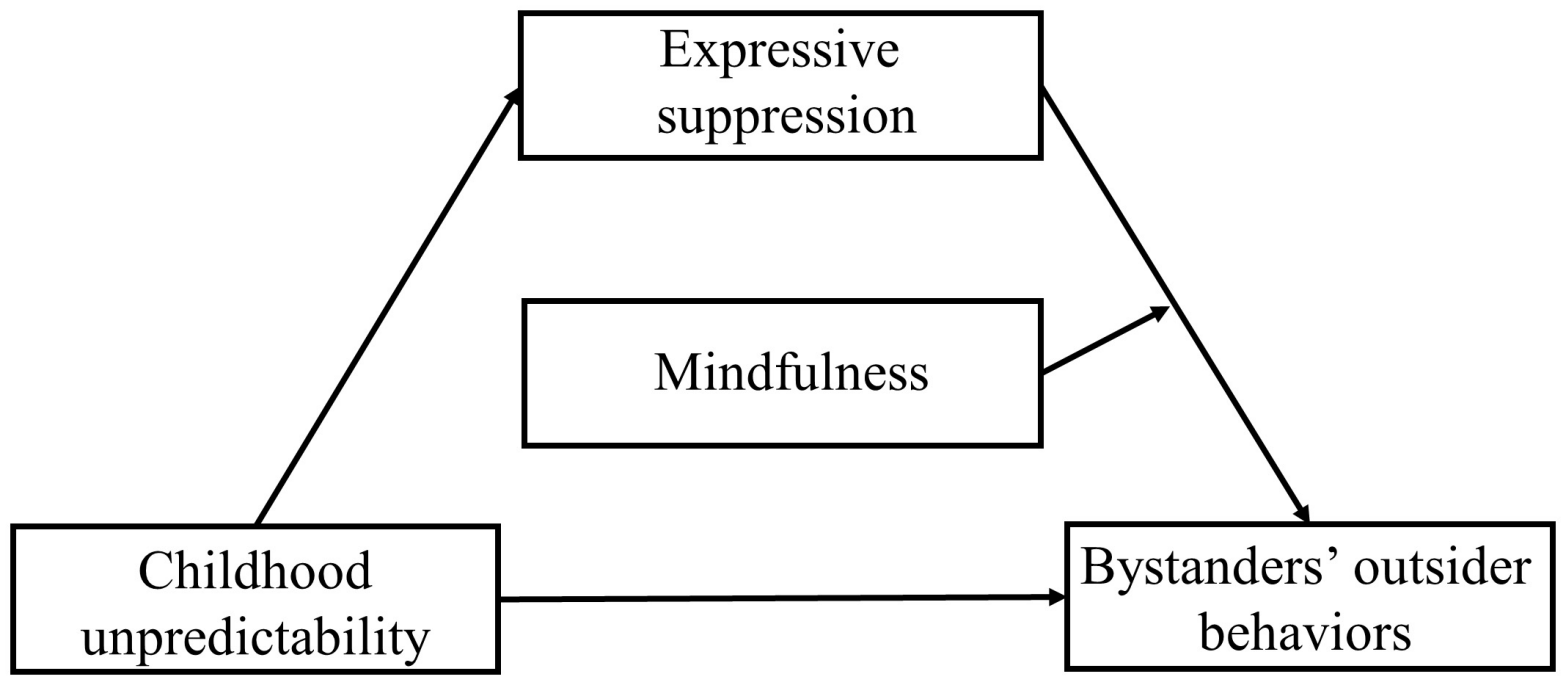
Proposed moderated mediation model.

In summary, the existing literature points to plausible connections between childhood unpredictability, expressive suppression, mindfulness, and bystander outsider behaviors in cyberbullying. However, key aspects remain to be fully explored and integrated. Specifically, prior research has not fully tested an integrated model that explains how these variables function together. This includes clarifying the mediating mechanism of expressive suppression and the potential buffering role of mindfulness within the context of undergraduate bystander behaviors. Addressing this gap is essential to advance beyond fragmented findings and to build a comprehensive framework for effective intervention. To address this, the present study employs a cross-sectional design and structural equation modeling (SEM) to examine three core pathways: (1) the direct association between childhood unpredictability and bystanders' outsider behaviors in cyberbullying among undergraduates, (2) the mediating role of expressive suppression in the relationship between childhood unpredictability and bystanders' outsider behaviors, and (3) the moderating role of mindfulness on the association between expressive suppression and bystanders' outsider behaviors. The conceptual framework is presented in Figure 1.

## 2 Materials and methods

### 2.1 Research design and ethics

This study employed a cross-sectional design to examine the relationships among childhood unpredictability, expressive suppression, mindfulness, and bystanders' outsider behaviors in cyberbullying among Chinese undergraduates. This research was approved by the Medical Research Ethics Committee of Hubei University of Chinese Medicine (Approval No. 20250169b). Informed consent was obtained from all individual participants included in the study. Prior to data collection, participants were presented with a detailed information sheet explaining the purpose of the study, the voluntary nature of participation, and the anonymity and confidentiality of their responses. They were explicitly informed of their right to withdraw from the study at any time without any penalty or negative consequence.

### 2.2 Participants

This study targeted undergraduates, a demographic considered vulnerable to unpredictable childhood environment, emotional issues, and cyberbullying (Kwan et al., 2020; Huang et al., 2021; Guo and Xia, 2023; Qi et al., 2024). Participants were recruited via convenience sampling from universities in Wuhan, Chongqing, and Shenzhen. These cities were selected for their representative mid-tier economic and educational profiles in China. An a priori power analysis was conducted using G^*^Power 3.1 to determine the required sample size for the planned analyses. Assuming a medium effect size (*f*^2^ = 0.15), a significance level of α = 0.05, and a desired statistical power of 0.95, the results indicated that a minimum sample size of 617 participants would be necessary to detect the hypothesized effects. A total of 1,520 students were invited, and 1,326 students returned questionnaires, representing an 87.2% response rate. Prior to analysis, all 1,326 returned questionnaires were screened. First, 177 questionnaires were excluded based on the scoring rules and other problematic reasons (e.g., patterned or non-varied responses, an unreasonably short completion time within 3 min, and an incorrect answer to the attention-check question about the number of posts made by the bullies in the Weibo web interface), resulting in a final analytic sample of 1,149 valid responses. The valid sample included 522 males (45.4%) and 627 females (54.6%). The mean age of participants was 19.48 years old (*SD* = 2.79), with an age range of 18–24 years old. The participants comprised 205 freshmen (17.8%), 417 sophomores (36.3%), 311 juniors (27.1%), and 216 seniors (18.8%). This distribution represented a cross-section of students from all four undergraduate years.

### 2.3 Data collection procedures

Data were collected anonymously online using the Wenjuanxing platform in April 2025. The study was conducted by trained psychology researchers who completed a standardized training program. Following Shen et al. (2024) approach to investigating bystanders' responses upon witnessing cyberbullying, a screenshot depicting a simulated cyberbullying event within a Weibo web interface was presented to participants as part of the questionnaire. Participants were instructed to carefully observe the incident and to envision themselves as an online user within this group. Then, participants were asked to complete the measures on the variables investigated in this study. To verify whether participants watched the information in the screenshot carefully, they were required to select the exact number of posts made by the bullies in the Weibo web interface. Participants were given sufficient time to reflect on their responses to ensure the data's accuracy. The questionnaire focused on the variables investigated in the study, which included childhood unpredictability, expressive suppression, mindfulness, and bystanders' outsider behaviors in cyberbullying. Demographic information, including gender, age, grade level, and university location, was also collected. The measurement tools were systematically adapted from validated scales. This adaptation involved a standardized forward-backward translation process and review by an expert panel for all non-native instruments, ensuring both conceptual fidelity and cultural relevance. Final approval of the questionnaires was obtained from three psychology experts to verify the appropriateness and safety of the content for all participants.

### 2.4 Measures

#### 2.4.1 Childhood unpredictability

Childhood unpredictability was measured using a 10-item scale adapted from Wang et al. (2022). This scale integrates items from Mittal et al. (2015), the Confusion, Hubbub, and Order Scale (Matheny et al., 1995), and the neighborhood subscale of People in My Life Questionnaire (Murray and Greenberg, 2006) to assess early-life environmental unpredictability. It assesses various aspects of an unpredictable childhood environment, including residential instability, parental inconsistency, household chaos, and neighborhood danger. Sample items include “People often moved in and out of my house on a pretty random basis” and “I often get drawn into other people's arguments at home.” Participants reflected on their experiences before age 10 and rated their agreement with each statement on a 5-point Likert scale (1 = *strongly disagree*, 5 = *strongly agree*). Higher scores indicated greater exposure to childhood unpredictability. The Cronbach's alpha value of this scale in this study was 0.81. A confirmatory factor analysis indicated that the scale demonstrated an acceptable fit with the data: χ^2^/*df* = 3.05, CFI = 0.91, TLI = 0.90, RMSEA = 0.07, and SRMR = 0.06.

#### 2.4.2 Expressive suppression

This variable was measured by the Expressive Suppression subscale from the Emotion Regulation Questionnaire (ERQ) developed by Gross and John (2003). This measurement assesses individuals' typical use of specific emotion regulation strategies. The expressive suppression subscale measures the tendency to inhibit the outward expression of emotions and consists of 4 items (e.g., “I keep my emotions to myself”). Participants rated their agreement with each statement on a 7-point Likert scale (1 = *strongly disagree*, 7 = *strongly agree*). Higher scores indicated a greater tendency to use expressive suppression. The Cronbach's alpha value of this scale in this study was 0.80. A confirmatory factor analysis indicated that the scale demonstrated an acceptable fit with the data: χ^2^/*df* = 3.02, CFI = 0.91, TLI = 0.90, RMSEA = 0.06, and SRMR = 0.05.

#### 2.4.3 Mindfulness

Mindfulness was assessed using the Chinese version (Liu et al., 2017) of the Child and Adolescent Mindfulness Measure (Greco et al., 2011). This instrument adopts a single-dimension structure comprising 10 items, each rated on a 5-point Likert scale (1 = *never*, 5 = *always*). It assesses one's contact with the present moment and acceptance of thoughts and feelings (e.g., “I stop myself from having feelings that I don't like” and “I think about things that happened in the past instead of thinking about things that are happening right now”). All items were reverse-scored, with higher total scores reflecting greater levels of mindfulness. The scale has been validated in adolescent and undergraduates, demonstrating good reliability and validity (Pepping and Duvenage, 2016; Yang et al., 2019). The Cronbach's alpha value of this scale in this study was 0.84. A confirmatory factor analysis revealed that the model fit indices were χ^2^/*df* = 2.95, CFI = 0.93, TLI = 0.92, RMSEA = 0.06, and SRMR = 0.05.

#### 2.4.4 Bystanders' outsider behaviors

Participants were required to give their behavioral responses using the measurement of the outsider behavior in cyberbullying developed by Shen et al. (2024). This questionnaire has 8 items including three dimensions: 3 items for self-disengagement, 3 items for cautious avoidance, and 2 items for victim blaming. Sample items include “I don't do anything because it's none of my business” and “Ignoring it because the victim is getting what they deserve.” Participants evaluated how accurately each item described their behaviors on a 7-point scale (1 = *strongly disagree*, 7 = *strongly agree*). Higher scores indicated higher levels of bystanders' outsider behavior in cyberbullying. The Cronbach's alpha value of this scale in this study was 0.87. A confirmatory factor analysis revealed that the model fit indices were χ^2^/*df* = 2.81, CFI = 0.93, TLI = 0.92, RMSEA = 0.06, and SRMR = 0.06.

### 2.5 Statistical analysis

The data analysis for this study was performed using SPSS 25.0 and Mplus 8.3 (Muthén and Muthén, 2009). Prior to the main analysis, the missing data in the valid questionnaires were carefully assessed, revealing a low item-level missing rate (under 5%). Consistent with recommended practices for structural equation modeling, Full Information Maximum Likelihood (FIML) estimation was used to address these missing values. FIML leverages all observed data to estimate model parameters, maintaining statistical power and producing unbiased estimates under the assumption that data are missing at random (MAR).

Subsequently, SPSS 25.0 was used for conducting common method variance and multicollinearity tests, descriptive statistics, and correlation analyses. Subsequently, SEM was employed in Mplus 8.3 to test the hypothesized relationships among childhood unpredictability, expressive suppression, mindfulness, and bystanders' outsider behaviors in cyberbullying. The SEM approach was chosen for its capacity to model complex pathways and simultaneously estimate direct, indirect, and moderating effects within a unified framework (Kline, 2023). Depending on the data's distribution, the model parameters were estimated using either Maximum Likelihood (ML) or Maximum Likelihood with Robust standard errors (MLR). In order to rigorously test for indirect effects, the bias-corrected bootstrapping method was applied, which offers robust statistical power without relying on normality assumptions (MacKinnon et al., 2002, 2004). Moderation effects were examined by including interaction terms in the model (Muthén and Muthén, 2009). Model fit was assessed using a combination of indices: χ^2^/*df* , CFI, TLI, RMSEA, and SRMR. A model was considered to have an acceptable fit if χ^2^/*df* was less than 5.0, CFI and TLI values were greater than or equal to 0.90, RMSEA and SRMR values were less than or equal to 0.08 (Hu and Bentler, 1999). In line with previous literature that identified gender and age as significant covariates (Schäfer et al., 2017; Jeyagobi et al., 2022; Wang et al., 2022; Lo, 2024), these demographic variables were included as statistical controls in all analyses.

## 3 Results

### 3.1 Common method bias and multicollinearity test

To address the potential for common method variance inherent in self-reported data, Harman's single-factor test was conducted (Podsakoff et al., 2003). The results revealed 13 factors with eigenvalues exceeding 1, with the first factor accounting for 27.09% of the total variance. This outcome suggests that common method bias was not a predominant concern in the dataset. Furthermore, multicollinearity was assessed by examining the variance inflation factor (VIF) and tolerance statistics for all predictors. The VIF values ranged from 1.18 to 2.03 (all below the recommended threshold of 5), and tolerance values fell between 0.49 and 0.85 (all above the recommended threshold of 0.1) (O'Brien, 2007). Therefore, common method bias and multicollinearity are unlikely to pose a substantial threat to the validity of the study's results.

### 3.2 Preliminary analysis

Descriptive statistics and correlation matrix for all study variables are reported in Table 1. As hypothesized, significant positive associations were observed between childhood unpredictability and expressive suppression (*r* = 0.21, *p* < 0.001) as well as bystanders' outsider behaviors in cyberbullying (*r* = 0.22, *p* < 0.001). Conversely, childhood unpredictability demonstrated a negative association with mindfulness (*r* = −0.20, *p* < 0.001). Additionally, Expressive suppression was negatively related to mindfulness (*r* = −0.41, *p* < 0.001) and positively related to bystanders' outsider behaviors in cyberbullying (*r* = 0.56, *p* < 0.001). Mindfulness was negatively related to bystanders' outsider behaviors in cyberbullying (*r* = −0.55, *p* < 0.001).

**Table 1:** Descriptive statistics and correlations between variables.

**Variables**	***M***	***SD***	**1**	**2**	**3**	**4**	**5**	**6**
1 Gender	—	—	—					
2 Age	19.48	2.79	−0.03	1				
3 CU	2.39	1.18	−0.04	−0.10^***^	1			
4 ES	3.57	1.95	−0.01	−0.03	0.21^***^	1		
5 Mindfulness	3.14	1.73	0.18^***^	−0.01	−0.20^***^	−0.41^***^	1	
6 BOBIC	3.02	1.86	−0.08^**^	0.10^***^	0.22^***^	0.56^***^	−0.55^***^	1

*N*= 1,149.

*M*, mean; SD, standard deviation; CU, childhood unpredictability; ES, expressive suppression; BOBIC, bystanders' outsider behaviors in cyberbullying. Gender was dummy coded with 0 for male participants and 1 for female participants.

^**^p < 0.01, ^***^p < 0.001.

It is noteworthy that while the correlation coefficients were significant, some of their absolute values were modest. This pattern is frequently observed in cyberbullying studies, as individual behaviors are shaped by a wide array of interacting factors. The obtained effects are consistent with Cohen's guidelines for a meaningful relationship in psychological research (Cohen, 1988; Kowalski et al., 2014). Despite their modest size, these significant correlations provide a foundational basis for proceeding with the proposed mediation and moderation analyses, which are designed to uncover the underlying mechanisms beyond simple bivariate links.

### 3.3 Measurement model

Before examining the structural model and testing the hypotheses, the measurement model was assessed to ensure adequate internal consistency reliability, convergent validity, and discriminant validity (Hair, 2011; Hair et al., 2017). This step involved evaluating the strength and significance of the relationships between observed indicators (measured items) and their corresponding latent constructs. As presented in Table 2, all constructs met the required thresholds for reliability and convergent validity. Consistent with Hair (2011), the reliability of individual items as indicators of their respective constructs was verified. Composite Reliability (CR) values for all constructs exceeded the recommended level of 0.70, demonstrating strong internal consistency across the measurement scales. Additionally, all items had R^2^ values above 0.36, confirming that the observed indicators adequately reflected their latent constructs. The Average Variance Extracted (AVE) for each construct was greater than 0.50, indicating that more than half of the variance in the observed variables was accounted for by the underlying construct rather than measurement error, thus supporting convergent validity.

**Table 2:** Reliability and convergent validity results.

**Construct**	**CR**	**AVE**	**Estimate range**	***R^2^* range**
CU	0.92	0.65	0.77–0.83	0.59–0.69
ES	0.83	0.59	0.72–0.80	0.52–0.64
Mindfulness	0.91	0.60	0.74–0.83	0.55–0.69
BOBIC	0.95	0.69	0.76–0.88	0.58–0.77

CU, childhood unpredictability; ES, expressive suppression; BOBIC, bystanders' outsider behaviors in cyberbullying; CR, composite reliability; AVE, average variance extracted.

To determine whether each construct captures a distinct concept and to identify any potential conceptual overlap, discriminant validity was examined using the Fornell-Larcker criterion (Hair et al., 2017). This method compares the square root of the Average Variance Extracted (AVE) for each construct with its correlations against other constructs. As shown in Table 3, the square root of the AVE for each construct, namely childhood unpredictability (0.80), expressive suppression (0.77), mindfulness (0.77), and bystanders' outsider behaviors in cyberbullying (0.83), was greater than its correlations with all other constructs. This confirms that each construct shares more variance with its own measures than with others, establishing discriminant validity and supporting the distinctiveness of the constructs within the measurement model.

**Table 3:** Discriminant validity results.

**Construct**	**Estimate range**	**CR**	**AVE**	**CU**	**ES**	**Mindfulness**	**BOBIC**
CU	0.77–0.83	0.92	0.65	**0.80**			
ES	0.72–0.80	0.83	0.59	0.26	**0.77**		
Mindfulness	0.74–0.83	0.91	0.60	−0.24	−0.49	**0.77**	
BOBIC	0.76–0.88	0.95	0.69	0.27	0.61	−0.58	**0.83**

CU, childhood unpredictability; ES, expressive suppression; BOBIC, bystanders' outsider behaviors in cyberbullying; CR, composite reliability; AVE, average variance extracted.

Diagonal elements (in bold) represent the square roots of AVEs.

### 3.4 Structural model

Upon establishing the reliability and validity of the measurement model, the structural model was examined to test the hypothesized relationships among constructs and evaluate the overall explanatory power of the theoretical framework (Hair et al., 2017). First, a baseline structural model without latent interaction terms was estimated. The model demonstrated acceptable fit to the data, with the following indices: χ^2^/*df* = 2.77, CFI = 0.93, TLI = 0.91, RMSEA = 0.05, and SRMR = 0.05, supporting its use for hypothesis testing. Next, a latent interaction term was introduced to examine whether model fit could be improved. Since the Latent Moderated Structural (LMS) approach in Mplus 8.3 employs a non-linear likelihood function and does not assume multivariate normality, conventional fit indices (χ^2^*/df* , CFI, TLI, RMSEA, and SRMR) are not available. Therefore, model selection was guided by information criteria, specifically the Akaike Information Criterion (AIC) and the Bayesian Information Criterion (BIC). In line with established practice, AIC was preferred over BIC for model comparison, as BIC tends to penalize model complexity more heavily, while AIC is asymptotically efficient in identifying a model that balances fit and parsimony (Yang, 2005). As summarized in Table 4, the model including the interaction term showed a lower AIC value (1,174.98) compared to the baseline model (1,182.20). This decrease in AIC suggests that incorporating the interaction enhanced the model's ability to represent the data without overfitting, offering preliminary support for the moderating role of mindfulness and warranting further interpretation of the interaction parameter.

**Table 4:** Model comparison based on AIC.

**Model**	**AIC**	**ΔAIC**
Baseline model	1,182.20	
Latent interaction model	1,174.98	−7.22

AIC, Akaike Information Criterion.

ΔAIC is calculated relative to the Baseline Model. A lower AIC indicates a better balance of model fit and parsimony.

### 3.5 Indirect and direct effects testing

To investigate potential underlying mechanisms, indirect pathways were examined using the product of coefficients method (MacKinnon et al., 2007; Hayes and Scharkow, 2013) with bias-corrected bootstrapping (5,000 resamples). All models controlled for participant age and gender. Age was significantly positively associated with bystanders' outsider behaviors (Estimate = 0.12, β = 0.11, *p* < 0.01), whereas gender was not significant (Estimate = −0.04, β = −0.03, *p* = 0.23). While the cross-sectional nature of this study precludes definitive causal inference, this analytical approach allows for the assessment of direct and indirect effects, thereby offering valuable insights into the proposed relationships. Expressive suppression was tested as a mediator between childhood unpredictability and bystanders' outsider behaviors in cyberbullying. As shown in Table 5, the path from childhood unpredictability to expressive suppression was statistically significant [Estimate = 0.24, β = 0.20, *p* < 0.001, 95% CI (0.19, 0.36)], indicating that greater childhood unpredictability was associated with higher expressive suppression. Similarly, expressive suppression was positively associated with bystanders' outsider behaviors in cyberbullying [Estimate = 0.46, β = 0.42, *p* < 0.001, 95% CI (0.38, 0.56)]. The indirect effect from childhood unpredictability to bystanders' outsider behaviors in cyberbullying through expressive suppression was also statistically significant [Estimate = 0.11, β = 0.08, *p* < 0.001, 95% CI (0.07, 0.18)], accounting for approximately 32.4% of the total effect and indicating partial mediation. These findings suggest the presence of a significant indirect association, whereby expressive suppression may serve as a potential pathway linking childhood unpredictability to bystanders' outsider behaviors in cyberbullying. Meanwhile, the direct effect from childhood unpredictability to bystanders' outsider behaviors in cyberbullying was also statistically significant [Estimate = 0.23, β = 0.20, *p* < 0.001, 95% CI (0.15, 0.32)]. Therefore, H1 and H2 were supported.

**Table 5:** Indirect and direct effects testing results.

**Path**	**Estimate**	**β**	**SE**	**Est./SE**	***p***	**Bias-corrected 95% CI**
CU → ES	0.24	0.20	0.04	5.98	< 0.001	[0.19, 0.36]
ES → BOBIC	0.46	0.42	0.04	11.53	< 0.001	[0.38, 0.56]
Indirect	0.11	0.08	0.02	5.52	< 0.001	[0.07, 0.18]
Direct	0.23	0.20	0.04	5.76	< 0.001	[0.15, 0.32]

CU, childhood unpredictability; ES, expressive suppression; BOBIC, bystanders' outsider behaviors in cyberbullying; SE, standard error; CI, confidence interval.

### 3.6 The moderating effect of mindfulness

In the moderated mediation model, age remained significantly positively associated with bystanders' outsider behaviors (Estimate = 0.10, β = 0.09, *p* < 0.05), while gender was non-significant (Estimate = −0.03, β = −0.02, *p* = 0.31). The moderating effect of mindfulness was examined in the relationship between expressive suppression and bystanders' outsider behaviors in cyberbullying. The results in Table 6 show that childhood unpredictability was significantly and positively associated with expressive suppression [Estimate = 0.24, β = 0.20, *p* < 0.001, 95% CI (0.19, 0.36)]. Expressive suppression was also significantly and positively associated with bystanders' outsider behaviors in cyberbullying [Estimate=0.39, β = 0.35, *p* < 0.001, 95% CI (0.33, 0.45)]. Meanwhile, the direct effect from childhood unpredictability to bystanders' outsider behaviors in cyberbullying was also statistically significant [Estimate = 0.21, β = 0.18, *p* < 0.001, 95% CI (0.13, 0.29)]. These results are consistent with the indirect and direct testing, which indicated that expressive suppression could potentially serve as a linking pathway between childhood unpredictability and bystanders' outsider behaviors in cyberbullying. Similarly, mindfulness was significantly associated with bystanders' outsider behaviors in cyberbullying [Estimate = −0.29, β = −0.27, *p* < 0.001, 95% CI (−0.35, −0.23)], and the interaction term between expressive suppression and mindfulness was also statistically significant [Estimate = −0.09, β = −0.08, *p* < 0.01, 95% CI (−0.15, −0.03)], suggesting that mindfulness may moderate the association between expressive suppression and bystanders' outsider behaviors in cyberbullying. Specifically, the negative direction of the interaction indicates that as undergraduates' mindfulness increased, the positive association between expressive suppression and bystanders' outsider behaviors in cyberbullying weakened. Therefore, H3 was supported.

**Table 6:** Hypothesis testing results.

**DV**	**IV**	**Estimate**	**β**	**SE**	**Est./SE**	***p***	**Bias-corrected 95% CI**
ES	CU	0.24	0.20	0.04	5.98	< 0.001	[0.19, 0.36]
BOBIC	CU	0.21	0.18	0.04	5.25	< 0.001	[0.13, 0.29]
BOBIC	ES	0.39	0.35	0.03	13.04	< 0.001	[0.33, 0.45]
BOBIC	Mindfulness	−0.29	−0.27	0.03	−9.67	< 0.001	[−0.35, −0.23]
BOBIC	Mindfulness × ES	−0.09	−0.08	0.03	−3.08	< 0.01	[−0.15, −0.03]

CU, childhood unpredictability; ES, expressive suppression; BOBIC, bystanders' outsider behaviors in cyberbullying; DV, dependent variable; IV, Independent variable; SE, standard error.

**Figure 2:**
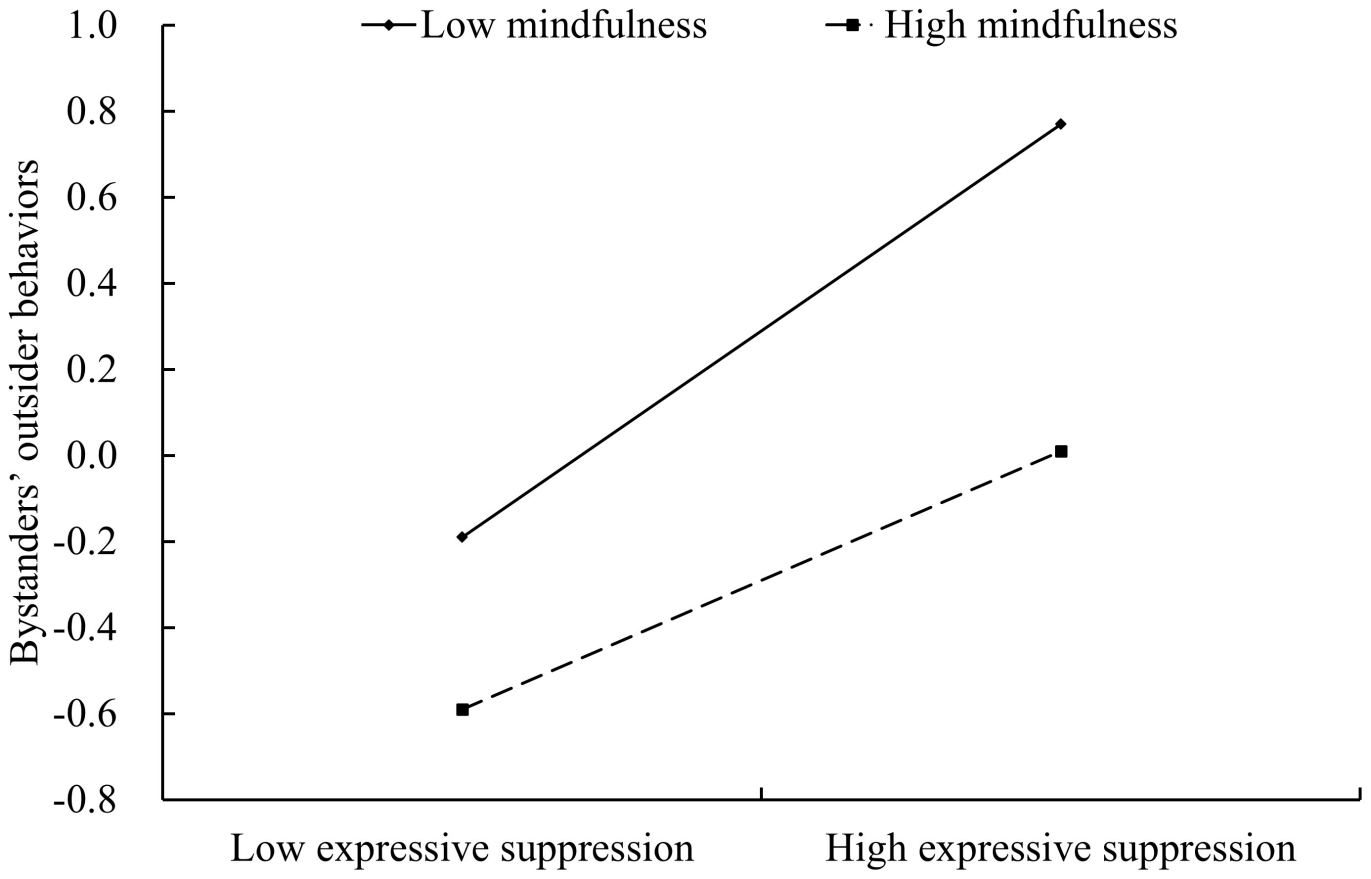
Mindfulness moderates the relationship between expressive suppression and bystanders' outsider behaviors in cyberbullying.

To further elucidate the moderating effect of mindfulness, simple slope tests were conducted in this study (Preacher et al., 2006). The simple slope tests indicated that the association between expressive suppression and bystanders' outsider behaviors in cyberbullying was positive at all levels of mindfulness, although the strength of the association varied. As shown in Figure 2, at a lower level of mindfulness (*M–*1*SD*), the association appeared relatively strong [*b*_*simple*_ = 0.48, *p* < 0.001, 95% CI (0.40, 0.56)]. At a higher level of mindfulness (*M* + 1*SD*), the association was weaker [*b*_*simple*_ = 0.30, *p* < 0.001, 95% CI (0.22, 0.38)]. These findings provide further evidence that the positive association between expressive suppression and bystanders' outsider behaviors in cyberbullying may weaken as undergraduates' mindfulness increases.

## 4 Discussion

Given the status of cyberbullying as a pressing public health concern among undergraduates, research efforts have intensified to elucidate its underlying mechanisms, with the role of bystanders identified as a critical area of inquiry (Gahagan et al., 2016; Doane et al., 2020; Jeyagobi et al., 2022). The association between early environmental risks and bystander behavior has received considerable empirical support (Feng et al., 2024; Li et al., 2024, 2025). However, research exploring how childhood unpredictability shapes undergraduates' outsider behavior in cyberbullying remains in its infancy. Thus, we formulated and examined a moderated mediation model based on existing literature to clarify the association between childhood unpredictability and bystanders' outsider behavior and to reveal the mediating role of expressive suppression and the moderating role of mindfulness. Our findings have both theoretical and practical significance. They enhance understanding of how childhood unpredictability relates to bystanders' outsider behavior among undergraduates and provide empirical support for developing intervention strategies from a bystander perspective.

### 4.1 Childhood unpredictability and bystanders' outsider behaviors in cyberbullying

The results revealed that childhood unpredictability is significantly and positively associated with bystanders' outsider behaviors in cyberbullying among undergraduates, thus validating H1. This finding aligns with life history theory, which posits that unpredictable early environments calibrate individuals toward faster life history strategies, prioritizing immediate safety over uncertain long-term social investments (Hill, 1993; Wang et al., 2022; Xu et al., 2023). Within the ambiguous context of cyberbullying, this strategic calibration manifests as a pattern of calculated disengagement. Bystanders opt for passive non-involvement to circumvent potential social risks and emotional costs (Szepsenwol et al., 2022; Qi et al., 2024; Li et al., 2025). This tendency is cognitively reinforced by a shift toward intuitive, low-effort decision-making under perceived threat (Wang et al., 2022). This automatic processing mode effectively supplants the more demanding, deliberative thinking required for proactive intervention (Doom et al., 2024). Furthermore, this disengagement is motivationally sustained. Childhood unpredictability fosters a stable tendency toward bystander inaction, marked by reduced social responsibility and low perceived intervention efficacy (Lindert et al., 2022; Topel et al., 2023; Xu et al., 2023; Park et al., 2024). This tendency results in a reliance on intuitive judgment combined with low behavioral motivation, thereby reinforcing the perception that intervention is futile. Consequently, their past experiences of unpredictability shape a cohesive cognitive-behavioral profile (Szepsenwol et al., 2015). This profile leads them to default to inaction in the face of social ambiguity, thereby reinforcing the outsider role in cyberbullying incidents.

This cognitive-motivational profile is further compounded by chronic emotional adaptations. Individuals exposed to childhood unpredictability often develop emotional blunting as a protective mechanism (Teicher et al., 2016; Milojevich et al., 2020). This blunting diminishes empathic concern for others' distress, resulting in attenuated emotional responsiveness to the harm caused by cyberbullying (McLaughlin et al., 2020). Such reduced empathic arousal may weaken their motivation to intervene when witnessing cyberbullying, as the emotional impetus for prosocial action is diminished (Eisenberg et al., 2016). The online environment systematically amplifies this predisposition. Anonymity and reduced social presence in cyberspace create a disinhibition effect (Chen, 2024; Knauf and Eschenbeck, 2025). For those already calibrated to avoid social risks, the attenuated fear of judgment online further lowers the threshold for passive behavior (Szepsenwol et al., 2022; Doom et al., 2024; Qi et al., 2024; Li et al., 2025). Thus, the convergence of early unpredictable experiences, a cognitive-motivational orientation toward risk minimization, and emotionally blunted responses fosters a stable behavioral profile characterized by outsider behavior in cyberbullying situations. Our findings extend life history theory by illustrating how early environmental unpredictability becomes biologically embedded and is situationally activated in cyberspace contexts. This contributes to the maintenance of the outsider role through default patterns of calculated disengagement.

### 4.2 Expressive suppression as a mediator

Results indicate that expressive suppression partially mediates the association between childhood unpredictability and bystanders' outsider behaviors in cyberbullying, supporting Hypothesis 2. This finding aligns with life history theory and the modal model of emotion regulation, suggesting that childhood unpredictability extends to encompass a critical emotional adaptation strategy, expressive suppression (Turliuc et al., 2020; Meyer et al., 2025). Conceptually, childhood unpredictability serves as a distal predictor of outsider behaviors, whereas expressive suppression functions as a more proximal mechanism directly involved in the manifestation of these responses. Thus, childhood unpredictability is both directly and indirectly associated with bystanders' outsider behaviors in cyberbullying through expressive suppression. This study thereby advances understanding of the mediating mechanism linking childhood unpredictability to bystanders' outsider behaviors among undergraduates.

Our finding of a positive association between childhood unpredictability and expressive suppression aligns with prior research (Schäfer et al., 2017; Gross and Cassidy, 2019; Szepsenwol et al., 2022; Li et al., 2025). This consistency is conceptually grounded in the perspective that early life adversity deviates from the expectable environment, a view mechanistically elaborated by the adaptive calibration model (Hill, 1993; Wade et al., 2022; Warmingham et al., 2023; Radley and Herman, 2023; Nelson et al., 2025). This model posits that exposure to early unpredictability orients behavioral and regulatory systems toward short-term priorities, favoring immediate emotional control over long-term planning. Within this adaptive framework, expressive suppression is conceptualized as a strategic regulatory adaptation shaped by early environmental conditions, rather than merely a habitual response (Warmingham et al., 2023). Inhibiting outward emotional expression serves to mitigate interpersonal risks in volatile caregiving environments, thereby reinforcing its function as a safety-enhancing mechanism (English and John, 2013). Longitudinal evidence substantiates this developmental pathway, showing that household chaos, a core marker of unpredictability, predicts increased reliance on suppression during adolescence (Meyer et al., 2025). Critically, such environments restrict opportunities for developing more adaptive, cognitive-oriented regulation strategies such as reappraisal, as unpredictable contexts fail to systematically reinforce reflective, long-term coping efforts (Young et al., 2019; Chu et al., 2023; Buthmann et al., 2024). This developmental process culminates in the entrenchment of suppression as a dominant regulatory phenotype that persists into adulthood (Hong et al., 2018).

Moreover, the observed association between greater expressive suppression and more frequent outsider behaviors is supported by the modal model of emotion regulation and aligns with the broader understanding of how emotion regulation affects social functioning (Aldao et al., 2010; Meyer et al., 2025). As demonstrated by Turliuc et al. (2020), emotion regulation strategies, particularly the underutilization of adaptive strategies, play a critical role in emotional responses to cyberbullying scenarios. The active inhibition of emotional expression consumes limited attentional resources and compromises the interpretation of socio-emotional cues (Binder et al., 2012; Da Silva Gomes et al., 2024). Expressive suppression, as a response-focused strategy, requires continuous monitoring and inhibitory control, thereby drawing upon mental resources needed for empathy, threat discrimination, and prosocial decision-making (Charles and Carstensen, 2008; Iacono et al., 2015; Chu and Carstensen, 2023). Consequently, undergraduates who rely heavily on expressive suppression exhibit reduced empathetic responsiveness and a weakened ability to accurately discern the distress signals of cyberbullying victims (Weissman et al., 2019; Chu et al., 2023; Kuhlman et al., 2023; Buthmann et al., 2024). This socio-emotional impairment manifests as diminished sensitivity to situational urgency and reduced feelings of personal responsibility, fostering the emotional disconnection and hesitancy characteristic of outsider behaviors (Chervonsky and Hunt, 2017; Ye et al., 2024). Thus, they develop emotional disengagement, diminished empathic concern, and reluctance to initiate intervention, consistent with the social disengagement functions of suppression (Ye et al., 2024; Meyer et al., 2025). In summary, our findings provide preliminary support for a specific mechanistic role of expressive suppression in clarifying the pathway from childhood unpredictability to bystander outsider behaviors in cyberbullying among undergraduates.

### 4.3 Mindfulness as a moderator

This study is among the first to examine the moderating role of mindfulness in the indirect association between childhood unpredictability and bystanders' outsider behaviors in cyberbullying. Our results demonstrated that mindfulness moderated the mediating effect of expressive suppression. Specifically, the indirect pathway from childhood unpredictability to bystanders' outsider behaviors via expressive suppression weakened as mindfulness levels increased, confirming Hypothesis 3. These findings align with the buffering hypothesis of mindfulness from a resource allocation perspective (Hölzel et al., 2011; Maxfield, 2020). This perspective posits that mindfulness frees cognitive capacity, thereby serving as a protective factor that attenuates the predictive association between a risk factor and an outcome. In the current study, mindfulness functioned as a protective factor, whereas expressive suppression operated as a risk mechanism underlying outsider behaviors. The interaction between these factors indicates that mindfulness effectively buffered the mediating role of expressive suppression in linking childhood unpredictability to bystanders' outsider behaviors among undergraduates.

This process can be clarified by the cognitive model of anxiety-related processing biases advanced by Mogg and Bradley (2016), which posits that when individual's process emotional information, their internal representations compete for attentional resources. Early life unpredictability cultivates a vigilant processing mode that heightens sensitivity to threatening social cues, such as online conflict, and compromises the ability to disengage attention from them. This predisposes individuals to automatically deploy inflexible emotion regulation strategies, including expressive suppression (Young et al., 2018; Hostinar and Miller, 2019). Mindfulness practice fosters an intentional, non-judgmental awareness of present-moment experiences (Creswell, 2017; Guendelman et al., 2017). This decentered stance enables individuals to perceive thoughts and urges as transient mental phenomena rather than undeniable facts requiring suppression or immediate reaction (Wang et al., 2025). When witnessing cyberbullying, bystanders with higher mindfulness are better able to sustain present-moment attention and adopt an accepting attitude toward their internal states, including urges to suppress emotions triggered by uncertainty or fear, even when encountering ambiguous social cues that typically induce hesitation (Waasdorp et al., 2022; Zhao et al., 2025). They also tend to display enhanced emotional clarity and metacognitive ability to detach from automatic reaction patterns, such as expressive suppression, associated with passive bystander behaviors (Cooper et al., 2018; Leyland et al., 2019). In essence, mindful awareness reduces the likelihood of outsider behaviors by interrupting the habitual link between emotional distress and behavioral disengagement (Pearson et al., 2015).

In contrast, undergraduates with low mindfulness tend to experience cognitive fusion with negative internal experiences, such as social evaluation anxiety. This fusion increases their susceptibility to being overwhelmed by such experiences and makes them more likely to assume a passive outsider role in cyberbullying (Emirtekin et al., 2020; Prieto-Fidalgo et al., 2022). They struggle to observe distressing thoughts and feelings with non-judgmental awareness, which intensifies feelings of ineptitude when managing the complex social environment of cyberspace characterized by publicity and ambiguity (Valk et al., 2017; Lindsay et al., 2019). Accordingly, individuals with low mindfulness are more likely to automatically engage in maladaptive emotion regulation strategies, such as expressive suppression, when confronted with ambiguous online conflicts that may echo early adverse experiences (Polanin et al., 2022). This strategic avoidance directly increases their likelihood of assuming a passive outsider role (Peters et al., 2014; Sahib et al., 2023; Maftei and Niţu, 2024). Given that undergraduates are still developing their psychological and emotional regulatory capacities, they experience particular difficulty handling the internal conflict and distress inherent in witnessing cyberbullying, which ultimately impedes prosocial action (Bussey et al., 2015; Twenge et al., 2017; Polanin et al., 2022; Kasturiratna et al., 2025). Conversely, mindfulness enhances the ability to assess one's internal and external reality with greater clarity, free from the distortions of intensified emotional reactivity (Lindsay and Creswell, 2019; Wenzel et al., 2024; Chen, 2024; Knauf and Eschenbeck, 2025). Therefore, mindfulness-based interventions may enhance the emotional awareness and self-regulatory capacity of undergraduates who experience high levels of childhood unpredictability and associated expressive suppression. This approach may encourage them to adopt more adaptive strategies and attitudes, such as acceptance and cognitive reappraisal, thereby mitigating the stress and passive tendencies linked to negative early experiences and reducing the risk of assuming an outsider role in cyberbullying.

### 4.4 Implications

The current study revealed the psychological mechanism between childhood unpredictability and bystanders' outsider behaviors in cyberbullying among undergraduates. In summary, our findings are consistent with the view that bystander passivity in cyberspace may be a correlate of early adaptive calibration, and that this association may be modifiable by contemporary mindfulness. From a theoretical perspective, there has been a noticeable limitation in research examining childhood unpredictability as a key predictor of bystander behavior in cyberbullying (McLoughlin et al., 2021). This study extends the research by delving into the association between childhood unpredictability and bystanders' outsider behaviors in cyberbullying. Quantitative findings reveal that individuals reporting greater childhood unpredictability are significantly more likely to engage in bystanders' outsider behaviors, a relationship mediated by the use of expressive suppression. Furthermore, this study proposes a mechanistic model to elucidate the development of bystanders' outsider behaviors within digital contexts, with a specific focus on the pathway from early life unpredictability to behavioral inaction via cognitive-emotional mechanisms. This study further substantiates the pivotal mediating role of expressive suppression in this pathway, thereby validating the applicability of the cognitive model of anxiety-related processing biases to outsider bystander dynamics in online, digitally-mediated interactions. Additionally, prior research predominantly focused on personal factors, such as empathy or moral disengagement, as independent variables to examine their impact on individuals' behaviors as bystanders in cyberbullying. This study introduces a unique developmental perspective by tracing the origins of bystander inaction to early life experiences. The investigated model illuminates how childhood unpredictability, through the mechanism of expressive suppression and moderated by mindfulness, shapes bystanders' outsider behaviors in cyberbullying. This not only verifies the applicability of the cognitive model of anxiety-related processing biases to bystander dynamics in cyberspace but also clarifies how the pathway from childhood unpredictability to behavioral outcomes is moderated by an individual's level of mindfulness. Such an exploration is conducive to further clarifying the complex interplay between early life unpredictability and subsequent emotion regulation among bystanders, thereby contributing to a more nuanced theoretical framework for understanding the developmental origins of maladaptive online behaviors and informing interventions aimed at fostering prosocial digital engagement and safeguarding online wellbeing.

The practical implications of this study should not be overstated. First, it underscores the essential role of educators in developing and implementing strategies to reduce outsider behavior among undergraduates in cyberbullying. Existing research also demonstrates that individuals with adverse childhood experiences tend to rely on expressive suppression as a maladaptive emotion regulation strategy (Ye et al., 2024; Koss et al., 2025), which in turn inhibits proactive intervention in online bullying situations. Given that childhood unpredictability fosters a reliance on suppression, interventions must directly target this maladaptive pattern. Educators should therefore facilitate guided discussions within university programs. These discussions should aim to help students recognize tendencies toward expressive suppression and practice alternative strategies, such as cognitive reappraisal, when witnessing cyberbullying (Zhao et al., 2023). Moreover, educators should work to build bystanders' communicative competence and self-efficacy for intervention. By enhancing their skills in de-escalating conflict and offering support to victims, this approach addresses the behavioral inaction directly resulting from emotional suppression (Doane et al., 2020; Macaulay et al., 2022). Furthermore, this study provides new evidence clarifying the mediating role of expressive suppression in the link between childhood unpredictability and passive bystander behavior among undergraduates. These findings underscore the need to refine campus mental health programs by specifically targeting this regulatory mechanism. Interventions should aim to help undergraduates recognize and reduce their reliance on suppression, fostering alternative strategies like cognitive reappraisal to manage distress when witnessing cyberbullying, thereby promoting more adaptive and prosocial forms of digital engagement. Lastly, this study offers valuable insights for educational interventions aimed at cultivating mindfulness among undergraduates, enabling them to respond effectively to cyberbullying incidents, and presents a fresh approach for educators to address the widespread occurrence of cyberbullying by enhancing students' present-moment awareness. For example, preventative initiatives could encompass mindfulness-based practices and experiential exercises aimed at bolstering emotional regulation. These sessions would specifically highlight the cultivation of metacognitive awareness, such as recognizing automatic emotional reactions, developing distress tolerance, and fostering a non-judgmental stance toward internal experiences. Educators, by incorporating these mindfulness exercises within broader mental health programs and targeted anti-cyberbullying interventions, have the potential to not only enhance students' self-regulation capacities but also to inspire a shift toward more conscious and proactive bystander behaviors in digital spaces, ultimately contributing to safer online communities and the psychological wellbeing of all participants (Proeve et al., 2018).

### 4.5 Limitations and future directions

Several limitations of this study should be acknowledged. First, a primary limitation is the cross-sectional nature of the data, which precludes strong causal claims for the proposed model and leaves open the possibility that unmeasured variables could offer alternative explanations for the observed associations. Future research should employ longitudinal designs with extended time intervals and repeated measurements to establish temporal precedence and examine reciprocal dynamics among the constructs. Such an approach would provide stronger evidence for causal pathways and yield deeper insight into the motivational processes underlying bystander responses in cyberbullying. For instance, ecological momentary assessment could capture individuals' real-time use of expressive suppression when encountering ambiguous online social cues, thereby clarifying how momentary fluctuations in this regulatory strategy mediate the link between early adversity and subsequent bystander inaction as it naturally unfolds. Second, the sole use of self-report questionnaires constrains the present findings. The automatic nature of expressive suppression limits individuals' capacity to accurately introspect on its use, while bystander inaction may be underreported due to social desirability concerns. To overcome these limitations, future studies should adopt a multi-method framework incorporating direct behavioral observation in experimentally controlled online simulations alongside peer-reported assessments of participants' typical bystander responses. This approach would provide a more robust and ecologically grounded test of the proposed model. Third, the generalizability of our findings is constrained by the convenience sampling method and the restriction of participants to Chinese undergraduates. Cultural factors, such as collectivism rooted in Confucian traditions, may shape both the development of expressive suppression as a response to early adversity and the normative expectations regarding bystander intervention. For instance, in collectivistic cultures, emotional suppression is often encouraged as a means of maintaining social harmony, which may amplify the mediating role of expressive suppression in the link between childhood unpredictability and outsider behaviors. Conversely, in more individualistic cultural contexts, different emotion regulation strategies or bystander response patterns may emerge. Therefore, future cross-cultural studies are needed to examine whether the proposed pathway from childhood unpredictability to bystanders' outsider behaviors in cyberbullying holds across diverse sociocultural environments (Yang et al., 2017; Zhao et al., 2023). Such comparative research would not only test the boundary conditions of our model but also contribute to a more nuanced understanding of how cultural contexts shape the developmental and regulatory mechanisms underlying bystander behavior. Fourth, the use of a scale designed to capture the multi-dimensional characteristics of outsider behavior (e.g., passive observation, intentional avoidance) may not fully reflect the spontaneity of real-time reactions in actual cyberbullying incidents (Shen et al., 2024). Although this method allowed for a systematic assessment of non-intervention tendencies, its focus on measuring passive behavioral patterns meant it omitted the evaluation of potential positive interventions, such as defending the victim or reporting the incident. Consequently, the present findings offer a representation of outsider inaction but do not encompass the full spectrum of bystander-related behaviors. Future research should incorporate real-world behavioral data, such as digital traces from social media or controlled online experiments with confederate victims, to capture bystander responses more ecologically. Additionally, simultaneously assessing both passive and proactive intervention behaviors within the same design would help clarify whether the mechanisms identified in this study are specific to outsider inaction or also predict prosocial responses. Such an approach would advance theoretical understanding of bystander dynamics and inform interventions aimed at promoting constructive digital citizenship. Finally, although our study focuses specifically on cyberbullying, passive bystander responses are also prevalent in other forms of interpersonal harm, such as sexual violence (Park and Kim, 2023; Bouchard et al., 2024). Although the contexts differ markedly, it is theoretically plausible that early life adversity, such as childhood unpredictability, may similarly shape emotion regulation tendencies that underlie passive responses across settings. The developmental pathway identified in this study, in which childhood unpredictability increases reliance on expressive suppression and thereby heightens the likelihood of outsider behavior, may offer a framework applicable beyond online bullying. Future research could examine whether this same mechanism operates in offline bystander contexts, particularly where emotional avoidance or suppression is socially reinforced. Nevertheless, any such extension must account for fundamental differences between digital and physical environments, including anonymity, permanence, and audience dynamics, which may uniquely influence how early adversity translates into bystander inaction in cyberspace.

## 5 Conclusions

The study's results suggest that childhood unpredictability is significantly and positively associated with bystanders' outsider behaviors in cyberbullying among undergraduates. Furthermore, the mediation analysis indicates that expressive suppression may play a potential mediating role in this relationship. The moderated mediation analysis shows that mindfulness was observed to moderate the association between expressive suppression and bystanders' outsider behaviors in cyberbullying among undergraduates. In particular, the association between expressive suppression and bystanders' outsider behaviors in cyberbullying is greater for those with lower mindfulness than for those with higher mindfulness.

## Data Availability

The raw data supporting the conclusions of this article will be made available by the authors, without undue reservation.
