# Supplementary material for: Childhood unpredictability, expressive suppression, and mindfulness as predictors of bystanders' outsider behaviors in cyberbullying among Chinese undergraduates: a moderated mediation model
Source: Front Psychol. 2026 Mar 25;17:1798978. doi: 10.3389/fpsyg.2026.1798978 (PMC13057508; doi:10.3389/fpsyg.2026.1798978)
Supplement: Supplementary file 1 [file Supplementary_file_1.docx]

Supplementary Material

*Note: The following materials were presented to participants in Chinese. This English translation is provided for reference.*

**1 Informed Consent Form Instructions**

We are conducting a scientific research study on university students' online behaviors and psychological well-being. This study aims to understand young people's experiences and perceptions in the online environment, particularly their responses as bystanders when witnessing cyberbullying incidents. The findings will contribute to a better understanding of the relevant psychological mechanisms and provide a scientific basis for preventing and intervening in cyberbullying.

Thank you very much for considering participating in our study. This study adopts a questionnaire-based survey. If you agree to participate, we will first present you with a screenshot of a simulated cyberbullying scenario. Please imagine yourself as a member of this online group and observe the incident carefully. Then, you will be asked to complete a series of questions based on your genuine feelings and thoughts. The entire process will take approximately 25 minutes.

No payment is required for participation in this study. As a token of appreciation for your time and effort, you will receive a small gift (e.g., stationery) upon completion of all questionnaires.

Your responses will be used solely for this research. This study will not provide you with direct treatment or monetary benefits. However, your participation will help the scientific community and society better understand university students’ online behaviors and psychological well-being. The research findings may contribute to the development of more effective cyberbullying prevention and intervention programs for university students in the future, thereby benefiting more people. You will also have an opportunity to reflect more deeply on your own online behaviors and feelings.

Your privacy will be strictly protected. This study adopts an anonymous approach. You will not be required to provide your name, student ID number, or any other information that could directly identify you. Only a study number will be used to match your questionnaires. All information will be kept strictly confidential and used solely for overall scientific research analysis. No identifiable personal information will appear in any research reports or publications.

Your participation is completely voluntary. You have the right to withdraw from this study at any time, for any reason, without any penalty or adverse consequences. You may also skip any question you prefer not to answer. Your decision will not affect the gift you are entitled to receive.

*Participant’s Declaration:*

*The researcher has fully explained to me all the information regarding the study described above. I understand the purpose, procedures, potential risks, and benefits of this study. All of my questions have been answered to my satisfaction. I agree to allow my child to participate in this study, and I understand that my child has the right to withdraw at any time during the study.*

**2 Demographic Questions**

1. Grade: ________
2. Age: ________ years old
3. Gender: ① Male ② Female

**3.1 Cyberbullying Scenario and Attention Check**

| *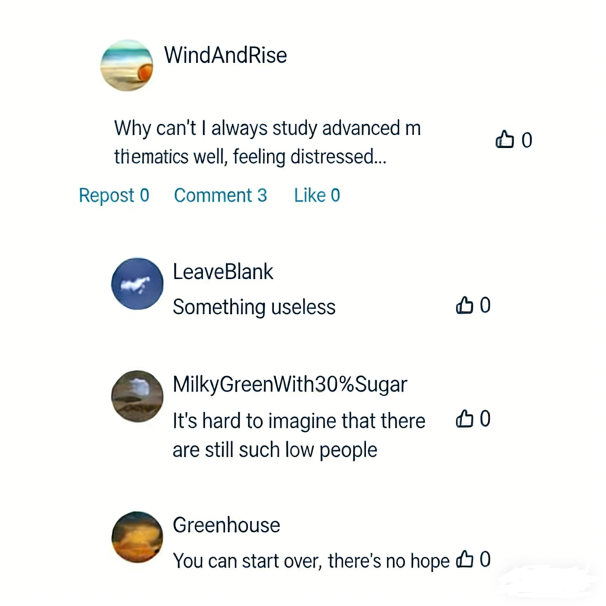* |
| --- |
| *Translation in English* |

*Please carefully observe the simulated cyberbullying incident presented in the screenshot below, which depicts a discussion on a Weibo web interface. Imagine yourself as an ordinary user viewing this discussion within this online group.*

In the screenshot you just viewed, how many posts (comments) did the bullies make in total ?

① 1 post; ② 2 posts; ③ 3 posts; ④ 4 posts; ⑤ 5 posts

**3.2 Childhood Unpredictability Questionnaire**

*Think back to your life when you were younger than 10 years old. This period includes preschool, kindergarten, and the first few years of elementary school. For each of the statements below, please indicate how much you agree or disagree that it describes your childhood experiences before age 10. There are no right or wrong answers, just respond based on what you remember.*

Please use the following scale: 1 = Strongly disagree; 2 = Disagree; 3 = Neutral; 4 = Agree; 5 = Strongly agree.

(1) Things were often chaotic in my house.

(2) People often moved in and out of my house on a pretty random basis.

(3) I had a hard time knowing what my parent(s) or other people in my house were going to say or do from day-to-day.

(4) I often get drawn into other people’s arguments at home.

(5) It is a real zoo in our home.

(6) There is often a fuss going on at our home.

(7) I feel scared in my neighborhood.

(8) Lots of kids in my neighborhood get into trouble.

(9) There are a lot of drugs and gangs in my neighborhood.

(10) My neighborhood is a dangerous place to live.

**3.3 Emotion Regulation Questionnaire (ERQ) -** **Expressive Suppression Subscale**

*We would like to ask you some questions about how you express your emotions. Specifically, the following items focus on your tendency to inhibit or suppress the outward expression of your feelings. Please read each statement carefully and indicate how well it describes your typical way of responding to emotions.* *There are no right or wrong answers, just respond based on what you feel.*

For each item, please answer using the following scale: 1 = Strongly disagree; 2 = Disagree; 3 = Somewhat disagree; 4 = Neutral; 5 = Somewhat agree; 6 = Agree; 7 = Strongly agree.

(1) I keep my emotions to myself.

(2) When I am feeling positive emotions, I am careful not to express them.

(3) I control my emotions by not expressing them.

(4) When I am feeling negative emotions, I make sure not to express them.

**3.4 The Child and Adolescent Mindfulness Measurement (CAMM)**

*We would like to ask you some questions about how you pay attention to your daily experiences and how you relate to your thoughts and feelings. Specifically, the following items focus on your tendency to be aware of what is happening in the present moment and to accept your thoughts and feelings without judgment. Please read each statement carefully and indicate how often each statement is true for you. There are no right or wrong answers, just respond based on what you really think or feel.* For each item, please answer using the following scale: 1 = Never; 2 = Rarely; 3 = Sometimes; 4 = Often; 5 = Always.

(1) I get upset with myself for having feelings that don’t make sense.

(2) At school, I walk from class to class without noticing what I’m doing.

(3) I keep myself busy so I don’t notice my thoughts or feelings.

(4) I tell myself that I shouldn’t feel the way I’m feeling.

(5) I push away thoughts that I don’t like.

(6) It’s hard for me to pay attention to only one thing at a time.

(7) I think about things that happened in the past instead of thinking about things that are happening right now.

(8) I get upset with myself for having certain thoughts.

(9) I think that some of my feelings are bad and that I shouldn’t have them.

(10) I stop myself from having feelings that I don’t like.

*Note for researchers: All 10 items of the Child and Adolescent Mindfulness Measure (CAMM) are negatively worded. In data analysis, all items were reverse-scored so that higher total scores reflect higher levels of mindfulness.*

**3.5 Bystanders’ outsider behaviors Measurement**

*Cyberbullying refers to acts in which someone intentionally harms, humiliates, or excludes others through online platforms using verbal attacks, insults, or other forms of aggression. While using social media in daily life, we may sometimes witness such incidents. Below are statements describing different ways people might respond when they witness cyberbullying. Please read each statement carefully and indicate how well it describes your actual thoughts or likely reactions, rather than how you think you should respond.*

For each item, please answer using the following scale: 1 = Strongly disagree; 2 = Disagree; 3 = Somewhat disagree; 4 = Neutral; 5 = Somewhat agree; 6 = Agree; 7 = Strongly agree.

(1) I don’t do anything because it’s none of my business.

(2) Avoiding the cyberbullying because I don’t care what happens.

(3) Ignoring it because I don’t want to get myself involved.

(4) I don’t do anything because I don’t know the full picture.

(5) Ignoring it because I don’t want to give the bully more attention.

(6) Avoiding the cyberbullying because I don’t want to make things worse for the victim.

(7) Ignoring it because the victim is getting what they deserve.

(8) I don’t do anything because the victim probably did something to cause it.
